# Trends in Prevalence, Awareness, Treatment, and Control of Hypertension in Rural Northeast China: 2008 to 2018

**DOI:** 10.1155/2020/1456720

**Published:** 2020-06-27

**Authors:** Liying Xing, Shuang Liu, Li Jing, Shuang Li, Yuanmeng Tian, Rui Zhang, Min Lin, Zhi Du, Dong Dai, Lei Shi, Guowei Pan

**Affiliations:** ^1^Institute of Preventive Medicine, China Medical University, Shenyang 110005, China; ^2^Department of Chronic Disease Preventive and Control, Liaoning Provincial Center for Disease Control and Prevention, Shenyang 110005, China; ^3^Department of Cardiovascular Ultrasound, The First Hospital of China Medical University, Shenyang 110005, China; ^4^Department of Cardiology, The Central Hospital of Benxi City, Benxi 117000, China; ^5^Department of Cardiology, The First Hospital of China Medical University, Shenyang 110005, China; ^6^Department of Chronic Disease Preventive and Control, Disease Control and Prevention of Dan Dong City, Dandong 118000, China; ^7^Department of Chronic Disease Preventive and Control, Disease Control and Prevention of Liao Yang City, Liaoyang 111000, China; ^8^Research Center for Universal Health, School of Public Health, China Medical University, Shenyang 110001, China

## Abstract

**Objective:**

This study is aimed at exploring the trends in the prevalence, awareness, treatment, and control of hypertension in rural northeast China from 2008 to 2018.

**Methods:**

Two successive cross-sectional surveys were conducted in Liaoning rural areas in 2008 and 2018, which included 131520 and 10926 representative participants aged ≥ 40 years, respectively.

**Results:**

Overall, the age-standardized prevalence of hypertension increased from 44.7% to 53.6%, and male residents showed a faster pace of increase and a 2.1-fold increase than female residents (25.5% vs. 10.6%) from 2008 to 2018. Moreover, the mean systolic and diastolic blood pressures increased by 9.0% and 4.1%, respectively, and the increase rates were greater in men than in women (9.2% vs. 8.9% and 5.3% vs. 3.5%, *P* < 0.05). Additionally, the prevalence of stage 2 and above hypertension was significantly higher in men than in women. However, the awareness, treatment, and control rates showed no improvement and remained unacceptably low. Control rates were 3.7% in 2008 and 3.6% in 2018. Even among individuals who received medical treatment, only 8.7% and 10.1% had controlled hypertension in 2008 and 2018, respectively.

**Conclusions:**

The prevalence of hypertension and mean blood pressure increased steadily in the past 10 years in rural northeast China, especially in men. However, the awareness, treatment, and control rates of hypertension remained extremely low. Therefore, long-term comprehensive strategies are urgently needed to prevent further development of cardiovascular diseases in these areas.

## 1. Introduction

Hypertension has long been considered as a major public health problem [1]. As a leading modifiable risk factor of various cardiovascular diseases, it accounts for nearly 54% of stroke and 47% of coronary artery disease [2]. A previous study reported that the number of individuals with hypertension was projected to be nearly 1.56 billion by 2025 worldwide, and approximately 3/4 of them will be in developing countries [3]. The economic burden of hypertension was estimated to cost approximately US $370 billion and 10% of healthcare expenditures worldwide [4]. Therefore, strategies targeted on hypertension management could provide underestimated public health gains.

The status of hypertension varies widely with different geographic, demographic, and socioeconomic factors. The prevalence of hypertension is significantly high in northeast China compared to those in other regions [5]. Moreover, more than half of the Chinese population is composed of rural residents. These populations possibly remain unaware, and hypertension remains untreated and uncontrolled despite the high prevalence [6]. Furthermore, rural China has been experiencing rapid epidemiologic transitions and economic progress in the past 10 years [7], so the status and distribution of hypertension might change accordingly. However, long-term estimates of the trends in hypertension remain scarce even though the prevalence of hypertension in different regions of China has been widely reported [8, 9].

An accurate estimation of the long-term trends of this condition is crucial in proposing effective strategies and programs regarding the prevention and control of hypertension, as well as planning the rational use of limited healthcare resources in rural China. In the present study, we conducted a cross-sectional survey of two large representative rural populations in 2008 and 2018, aiming to identify long-term natural changes in the prevalence, treatment, and control of hypertension in rural northeast China and further provide population-based evidence for formulating corresponding strategies.

## 2. Methods

### 2.1. Study Population

This consisted of two cross-sectional studies conducted in northeast rural China in 2008 and 2018. A multistage, stratified, and cluster random sampling method was used to ensure that the samples of the two studies were representative. Further details on the design are described as follows.

### 2.2. Cross-Sectional Study in 2008

**Figure 1:**
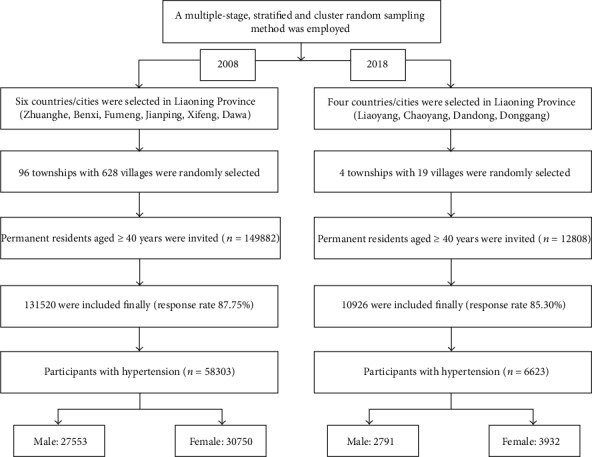
Flow chart of patient selection.

The study was conducted from September 2008 to November 2008. Six counties/cities (Zhuanghe city, Benxi county, Fumeng county, Jianping county, Xifeng county, and Dawa county) were selected from Liaoning Province. Subsequently, 70% of the township and 50% of the villages were randomly selected. Thereafter, 626 rural villages in 96 townships were randomly selected from these 6 counties/cities. All permanent residents aged ≥ 40 years in each village (*n* = 149882), except those who were pregnant or had a mental disorder, were eligible to participate; 131520 participants (87.75%) completed the study (Figure 1). The study was approved by the Central Ethics Committee of the Liaoning Provincial Center for Disease Control and Prevention. Written informed consent was obtained from all participants.

### 2.3. Cross-Sectional Study in 2018

The study was conducted between September 2017 and May 2018. Four counties (Chaoyang, Lingyuan, Liaoyang, and Donggang) were randomly selected from Liaoning Province. Subsequently, 19 rural villages were randomly selected from these four counties. All permanent residents aged ≥ 40 years in each village (*n* = 12808), except those who were pregnant or had a mental disorder, were eligible to participate; overall, 10926 participants (85.30%) completed the study (Figure 1). The study was approved by the Central Ethics Committee of the China National Center for Cardiovascular Disease. Written informed consent was obtained from all participants.

### 2.4. Measurements and Definitions

Patients and public will not be involved in the development of the research question or in the design of the study. Patients will receive written information about this trial. Moreover, the burden of hypertension will be assessed by investigators. After signing an informed consent by the participants, they will be assessed for eligibility and data collection will begin. Dissemination of the general results (no personal data) will be made on demand. For each participant, blood pressure was measured three times at 2 min intervals after at least 5 min of rest in the sitting position using a standardized automatic electronic sphygmomanometer (J30; Omron, Kyoto, Japan). Participants were asked during the interview whether they had used prescription medication for blood pressure in the past two weeks.

Hypertension was defined as a mean systolic blood pressure (SBP) ≥ 140 mmHg or a mean diastolic blood pressure (DBP) ≥ 90 mmHg and/or self-reported use of antihypertensive medication in the past 2 weeks according to the 2010 Chinese guidelines for the management of hypertension [10]. Prehypertension was considered as an SBP ≥ 120 mmHg and <140 mmHg and DBP ≥ 80 mmHg and <90 mmHg and not using antihypertensive medication. Stage 1 hypertension was defined as an average SBP ≥ 140 mmHg and <160 mmHg and/or DBP ≥ 90 mmHg and <100 mmHg; stage 2 hypertension was defined as SBP ≥ 160 mmHg and <180 mmHg and/or DBP ≥ 100 mmHg and <110 mmHg; stage 3 hypertension was defined as SBP ≥ 180 mmHg and/or DBP ≥ 110 mmHg [11].

Each participant had a medical history of hypertension. Awareness of hypertension was defined as having an answer of “Yes” to the question “Have you been diagnosed with hypertension by a certified doctor?” Treatment of hypertension was defined as use of antihypertensive medication in the past 2 weeks. Hypertension control was defined as an average SBP < 140 mmHg and an average DBP < 90 mmHg, while uncontrolled hypertension was considered as not meeting these criteria.

### 2.5. Statistical Methods

Descriptive statistics were calculated for all variables. Continuous variables with normal distribution are reported as means and standard deviations (SDs). Meanwhile, continuous variables are reported as medians and interquartile ranges. Student's *t*-test and nonparametric Mann–Whitney test were used, as appropriate, to compare differences in continuous variables between sex subgroups. All statistical analyses were conducted using SPSS 22.0 (SPSS Inc., Chicago, IL, USA). *P* values < 0.05 were considered statistically significant.

## 3. Results

### 3.1. Prevalence of Hypertension

In 2008, the sample included 131520 individuals with a mean age of 54.1 years (SD, 9.7), of which 64430 (48.9%) were men. On the contrary, in 2018, 10926 participants accomplished the study, with 4390 men (40.2%) and an average age of 60.0 years (SD 10.1).  Table 1 shows the prevalence of hypertension in the past 10 years. There was a noticeable increase in the prevalence of hypertension during this period. The crude prevalence of hypertension was 44.3% in 2008 and 60.6% in 2018. Noticeably, the prevalence of hypertension was lower in men than in women in 2008. However, it did not differ significantly between men and women in 2018 due to the steeper increase in the prevalence of hypertension in men. The highest age-specific prevalence of hypertension was observed in those aged 50–59 years in 2008; however, it changed into 60–69 years in 2018.

The age-standardized prevalence was 44.7% to 53.6% from 2008 to 2018, which increased by 16.5% during this period. Further, male residents showed a faster pace of increase than female residents, from 42.7% to 55.1% compared to 46.7% to 53.3%, respectively. The prevalence of hypertension in men showed a more rapid increase than that in women (29.0% vs. 14.1%, *P* < 0.05) in 10 years.

The percentages of normal condition and prehypertension showed a significant decrease in the past 10 years, especially in men, as shown in Table 2. However, the percentages of stage 2 and 3 hypertension in men increased significantly from 2008 to 2018, with rates of 44.3% and 19.0%, respectively.

### 3.2. Blood Pressure

The trends of blood pressure in the overall population were demonstrated in Table 3. From 2008 to 2018, these two groups manifested a continuous increase in both SBP and DBP. The mean SBP increased from 133.6 mmHg in 2008 to 145.7 mmHg in 2018, with a rate of 9.0% (9.2% in men and 8.9% in women). Meanwhile, the mean DBP increased from 83.3 mmHg to 86.7 mmHg, with a rate of 4.1% (5.3% in men and 3.5% in women). Additionally, SBP showed a gradual increase with increasing age in both 2008 and 2018; however, DBP did not show a significant change across all ages. In the population with hypertension, the SBP increased by 5.2% although DBP did not show a significant change.

### 3.3. Awareness, Treatment, and Control Rate of Hypertension

Overall, the awareness, treatment, and control rates of hypertension were 54.2%, 42.0%, and 3.7% in 2008 and 47.5%, 35.4%, and 3.6% in 2018 in northeast rural China (Table 4). The awareness and treatment of hypertension did not have a significant improvement during this period. Among those who are aware of their hypertension, 69.4% and 74.5% received medical treatment in 2008 and 2018, respectively. However, a majority of these individuals did not achieve better control of blood pressure, with low control rates of 8.7% and 10.1% in 2008 and 2018, respectively. What was worth noticeable was that, among those who did not achieve adequate control of blood pressure, stage 2 and 3 hypertension accounted for 47.1% and 46.4% in 2008 and 2018, respectively.

## 4. Discussion

The major findings of the study were as follows. (1) The age-standardized prevalence of hypertension increased from 46.0% to 53.6% in rural northeast China from 2008 to 2018, with a rate of 16.5%. (2) Men had higher increase rate in the prevalence of hypertension than women in the past 10 years. Moreover, the prevalence of stage 2 and 3 hypertension was higher in men than in women. (3) The SBP and DBP showed increasing trends in the overall population from 2008 to 2018, and greater upward shift of blood pressure in men than in women was observed, suggesting increased cardiovascular risk and end-organ damage in the future decades, especially in men. (4) Hypertension awareness, treatment, and control rates remained disproportionately low. Therefore, comprehensive strategies should be highlighted in the prevention and management of hypertension in terms of preventing adverse outcomes in the future decades.

The present study showed that the hypertension status was becoming worse over the past 10 years in rural northeast China. The prevalence of hypertension in rural northeast China was higher than the average national level conducted in 2002 and 2017 [8, 12], which also exceeded the estimation of 42% reported by the Prospective Urban Rural Epidemiology study in 2003–2009 [13]. Compared with other regions of China, the prevalence of hypertension obviously increased, with the frustrating low awareness, treatment, and control rates [14–16]. Compared to those in the studies conducted in other countries with no or slight changes in prevalence, awareness, treatment, and control rates, the status of rural northeast China remained suboptimal [1, 13, 15].

The awareness, treatment, and control rates of hypertension in rural north China increased significantly in 1991 to 2011 as previously reported. However, the two surveys were conducted in the same population [17]. On the contrary, the present study consisted of two representative samples without any intervention. In this regard, we considered that our study represented a more accurate estimation of natural changes during this period in rural northeast China. Liaoning Province has an overall population of 43.78 million, a majority of the residents are living in rural areas, and hypertension has become a great population burden and major public health challenge in these areas. Therefore, comprehensive strategies focusing on the prevention and treatment of hypertension in these areas should be emphasized.

The SBP showed a noticeable increase in the past 10 years in both overall population and those with hypertension, suggesting that the situation has markedly worsened over several years. As rural China has been experiencing rapid socioeconomic changes and health transitions [18, 19], the uncontrolled related risk factors including age, male sex, diabetes, obesity, dyslipidemia, hyperuricemia, and lifestyle change might largely contribute to the increasing burden of hypertension, although great efforts have been made to address the cause of hypertension and implement appropriate treatment. Moreover, it was not surprising that the prevalence of hypertension in male residents had a faster pace of increase. Male residents were likely to have multiple risk factors including smoking and drinking in rural areas [20]. Moreover, the age-specific prevalence was different from 2008 to 2018. The highest age-specific prevalence of hypertension was observed in those aged 50–59 years in 2008; however, it changed into 60–69 years in 2018. Longer life expectancy likely attributed to the change.

In addition to the increased prevalence, our study identified that the overall awareness, treatment, and control rates of hypertension remained less than 50%, 40%, and 7%, respectively, in the last decade. Compared to those in economically developed countries, they were far from satisfactory [4, 21, 22]. Moreover, the treatment and control rates of hypertension were still remarkably below the national average levels and those in other developing countries [8, 23, 24]. The persistent frustrating awareness, treatment, and control rates of hypertension suggested that aggressive education and screening were urgently required in these areas. Moreover, provision of adequate treatment and affordable medications provided by protocols and policies might be of great importance.

The disappointing awareness, treatment, and control rates indicated that a considerably large number of individuals with uncontrolled hypertension largely contributed to the high prevalence of stroke in northeast China, which elicited a major concern in these areas [6, 20]. Individuals with stage 2 and 3 hypertension were more likely to be aware of, treated for, and controlled for hypertension, although there were still a large proportion of this population who had uncontrolled hypertension [8, 25]. It was possible that individuals with low income and without prior cardiovascular events and/or coexisting conditions were less likely to seek medical attention [8]. Therefore, available and affordable therapies in addition to persistent education and long-term screening in the rural Chinese population were reasonably expected.

The strength of this study is that the surveys were conducted in two representative rural populations and were adequately powered, thus providing opportunity to assess the long-term natural changes of hypertension in rural northeast China. There are still several limitations in the study. First, the current hypertension guideline defined stage 1 hypertension as SBP of 130–139 mmHg or DBP of 80–89 mmHg [26]. However, in the present study, hypertension was defined according to the previous guideline, since the current Chinese guidelines for the management of hypertension recommended a blood pressure treatment goal of 140/90 mmHg [16, 27]. Second, we did not obtain information regarding the risk factors of hypertension, including obesity, diabetes, dyslipidemia, and lifestyle change, since it was well established recently [28, 29].

## 5. Conclusion

We provided the long-term natural evolution of the prevalence, awareness, treatment, and control of hypertension by conducting two cross-sectional studies in different representative samples. A comprehensive assessment of these changes was essential in exploring effective strategies in northeast rural China. The prevalence of hypertension dramatically increased in the past 10 years. The increasing number of individuals with hypertension and increased blood pressure has become a serious public problem in these areas, especially in men. Moreover, the awareness, treatment, and control rates were frustratingly low in each period, even among individuals with stage 2 and above hypertension. Inadequate management of the population with hypertension might have substantial economical and health consequences, including the development of cerebrovascular, kidney, and heart diseases. Therefore, our study highlighted the urgent need to develop comprehensive strategies for adequate management of hypertension in rural northeast China.

## Figures and Tables

**Table 1:** Prevalence and age-standardized prevalence rates of hypertension and their percentage change by sex in rural northeast China, 2008-2018.

Age groups (years)	Men			Women			Total			*P* for men	*P* for women	*P* for total
	2008	2018	Change (%)	2008	2018	Change (%)	2008	2018	Change (%)			
40-49	29.3	45.1	54.0	29.8	35.4	18.8	29.6	38.8	31.1	<0.001	<0.001	<0.001
50-59	43.3	54.0	24.8	48.0	53.8	12.1	45.7	53.9	17.9	<0.001	<0.001	<0.001
60-69	55.3	64.8	17.1	61.8	70.1	13.4	58.5	67.8	15.9	<0.001	<0.001	<0.001
70-79	66.8	77.3	15.7	71.1	80.4	13.0	68.8	78.9	14.7	<0.001	<0.001	<0.001
≥80	69.1	74.3	7.5	75.4	82.7	9.6	72.2	78.9	9.2	0.224	0.05	0.019
Total	42.8	61.3	43.2	45.8	60.2	31.4	44.3	60.6	36.8	<0.001	<0.001	<0.001
ASR∗	42.7	55.1	29.0	46.7	53.3	14.1	44.7	53.6	19.9			

**Table 2:** Prevalence of normal, prehypertension, stage 1, stage 2, and stage 3 and their percentage change by sex in rural northeast China, 2008-2018.

Age groups (years)	Men			Women			Total			*P* for men	*P* for women	*P* for total
	2008	2018	Change (%)	2008	2018	Change (%)	2008	2018	Change (%)			
Normal												
40-49	19.3	14.6	-24.0	33.9	28.6	-15.7	26.9	23.7	-11.7	0.003	<0.001	0.003
50-59	15.6	12.1	-22.6	21.8	14.2	-34.9	18.8	13.4	-28.7	0.001	<0.001	<0.001
60-69	13.0	9.1	-29.8	14.5	9.1	-37.4	13.8	9.1	-33.8	<0.001	<0.001	<0.001
70-79	10.1	4.0	-60.5	10.0	8.4	-16.1	10.1	6.4	-36.8	<0.001	0.14	<0.001
≥80	8.9	5.6	-37.7	7.4	4.0	-44.9	8.1	4.7	-41.9	0.192	0.128	0.042
Total	15.9	9.8	-38.4	23.9	14.1	-41.0	20.0	12.4	-38.0	<0.001	<0.001	<0.001
ASR∗	15.9	11.6	-27.0	23.5	18.0	-23.4	19.9	15.8	-20.6			
Prehypertension												
40-49	52.8	40.5	-23.1	37.6	37.1	-1.2	44.9	38.3	-14.6	<0.001	0.745	<0.001
50-59	42.6	36.0	-15.3	32.1	34.4	7.3	37.1	35.0	-5.7	<0.001	0.028	0.015
60-69	33.6	28.4	-15.5	25.8	23.6	-8.2	29.7	25.7	-13.7	<0.001	0.036	<0.001
70-79	25.2	20.2	-20.0	20.8	14.7	-29.4	23.1	17.2	-25.5	0.003	<0.001	<0.001
≥80	24.1	21.5	-10.7	18.6	16.2	-12.8	21.4	18.6	-12.9	0.519	0.48	0.29
Total	42.8	30.7	-28.3	32.0	28.2	-11.9	37.3	29.2	-21.7	<0.001	<0.001	<0.001
ASR∗	42.9	34.6	-19.3	31.4	30.8	-1.9	37.0	32.3	-12.7			
Stage 1												
40-49	18.5	27.6	49.5	16.7	22.5	34.9	17.5	24.3	38.5	<0.001	<0.001	<0.001
50-59	24.1	29.4	21.7	23.3	27.2	16.6	23.7	28.0	18.1	<0.001	<0.001	<0.001
60-69	27.8	31.5	13.1	26.4	31.6	19.5	27.1	31.5	16.2	0.002	<0.001	<0.001
70-79	30.8	34.9	13.2	27.1	28.2	4.4	29.0	31.3	7.9	0.024	0.468	0.059
≥80	31.5	34.0	8.0	25.3	27.2	7.2	28.4	30.3	6.5	0.564	0.634	0.528
Total	23.5	31.0	31.9	21.7	27.9	28.6	22.6	29.2	29.2	<0.001	<0.001	<0.001
ASR∗	23.3	29.7	27.5	21.7	26.2	20.7	22.5	27.5	22.2			
Stage 2												
40-49	6.2	12.2	95.6	7.8	8.2	4.2	7.1	9.6	35.5	<0.001	0.673	<0.001
50-59	11.6	16.2	39.4	14.3	16.9	17.9	13.0	16.6	27.7	<0.001	0.002	<0.001
60-69	16.9	21.6	27.6	20.6	21.9	6.3	18.7	21.7	16.1	<0.001	0.17	<0.001
70-79	21.5	25.5	18.8	24.4	29.4	20.2	22.9	27.6	20.5	0.012	0.002	<0.001
≥80	19.7	22.9	16.1	27.9	29.5	5.8	23.8	26.5	11.5	0.401	0.681	0.319
Total	11.6	19.4	67.2	13.9	18.9	36.0	12.8	19.1	49.2	<0.001	<0.001	<0.001
ASR∗	11.5	16.6	44.3	14.5	16.1	11.0	13.0	16.1	23.8			
Stage 3												
40-49	3.3	5.0	52.3	4.0	3.6	-9.4	3.6	4.1	12.3	0.015	0.515	0.31
50-59	6.1	6.3	3.8	8.6	7.4	-13.9	7.4	7.0	-5.3	0.744	0.062	0.41
60-69	8.7	9.5	9.0	12.7	13.8	8.7	10.7	12.0	12.1	0.297	0.153	0.017
70-79	12.3	15.4	24.8	17.7	19.3	9.0	14.9	17.5	17.5	0.019	0.256	0.007
≥80	15.7	16.0	1.4	20.9	23.1	10.7	18.3	19.9	8.6	0.948	0.536	0.527
Total	6.2	9.2	48.4	8.4	10.8	28.6	7.3	10.2	39.7	<0.001	<0.001	<0.001
ASR∗	6.3	7.5	19.0	8.9	8.9	0.0	7.6	8.2	7.9			

**Table 3:** Level of SBP and DBP (mmHg) in adults of rural northeast China by age and sex between 2008-2018.

Age group (years)	Men			Women			Total			*P* for men	*P* for women	*P* for total
	2008	2018	Change (%)	2008	2018	Change (%)	2008	2018	Change (%)			
SBP in adults												
40-49	127.1	134.6	5.9	125.2	130.3	4.1	126.1	131.8	4.5	<0.001	<0.001	<0.001
50-59	133.1	140.9	5.8	134.9	142.1	5.3	134.0	141.6	5.7	<0.001	<0.001	<0.001
60-69	139.0	147.9	6.4	143.1	151.8	6.1	141.0	150.1	6.4	<0.001	<0.001	<0.001
70-79	144.8	155.3	7.2	149.5	158.8	6.2	147.0	157.2	6.9	<0.001	<0.001	<0.001
≥80	146.9	154.5	5.2	152.5	162.0	6.2	149.7	158.6	6.0	0.001	<0.001	<0.001
Total	133.2	145.4	9.2	134.0	145.9	8.9	133.6	145.7	9.1	<0.001	<0.001	<0.001
DBP in adults												
40-49	81.2	88.0	8.4	79.6	83.7	5.1	80.3	85.2	6.0	<0.001	<0.001	<0.001
50-59	84.1	88.9	5.8	83.9	86.4	3.0	84.0	87.3	4.0	<0.001	<0.001	<0.001
60-69	85.4	87.6	2.6	86.1	86.6	0.6	85.8	87.0	1.5	<0.001	0.137	<0.001
70-79	86.6	87.2	0.7	87.1	85.3	-2.1	86.9	86.2	-0.8	0.265	<0.001	0.065
≥80	86.4	86.4	0.0	88.1	88.1	-0.1	87.3	87.3	0.1	0.977	0.971	0.96
Total	83.5	87.9	5.3	83.0	85.9	3.5	83.3	86.7	4.1	<0.001	<0.001	<0.001

**Table 4:** Awareness, treatment, and control of hypertension among rural adults of northeast China by age and sex in 2008-2018.

Age groups (years)	Men			Women			Total			*P* value for men	*P* value for women	*P* value for total
	2008	2018	Change (%)	2008	2018	Change (%)	2008	2018	Change (%)			
Awareness												
40-49	38.6	36.5	-5.4	49.5	40.6	-17.8	44.3	39.0	-12.0	0.468	<0.001	0.005
50-59	48.7	40.0	-17.9	59.6	48.6	-18.4	54.6	45.4	-16.9	<0.001	<0.001	<0.001
60-69	55.2	42.7	-22.7	64.5	52.6	-18.5	60.1	48.6	-19.1	<0.001	<0.001	<0.001
70-79	56.6	43.6	-23.0	64.0	59.9	-6.4	60.2	52.5	-12.8	<0.001	0.039	<0.001
≥80	52.5	37.4	-28.8	59.0	60.8	3.2	55.8	50.8	-9.0	0.006	0.698	0.166
Total	48.9	41.3	-15.6	58.9	51.7	-12.1	54.2	47.5	-12.3	<0.001	<0.001	<0.001
Treatment												
40-49	28.2	21.6	-23.5	37.3	27.7	-25.7	33.0	25.2	-23.5	0.013	<0.001	<0.001
50-59	36.0	25.5	-29.1	46.9	37.5	-20.1	42.0	33.1	-21.2	<0.001	<0.001	<0.001
60-69	42.7	31.3	-26.8	52.0	42.8	-17.8	47.6	38.1	-19.9	<0.001	<0.001	<0.001
70-79	44.5	29.4	-33.8	52.5	49.1	-6.4	48.4	40.2	-16.9	<0.001	0.103	<0.001
≥80	42.3	15.9	-62.4	47.2	38.5	-18.5	44.8	28.8	-35.8	<0.001	0.073	<0.001
Total	37.0	27.8	-24.8	46.5	40.6	-12.7	42.0	35.4	-15.7	<0.001	<0.001	<0.001
Control												
40-49	4.5	0.7	-85.0	4.5	3.2	-27.4	4.5	2.2	-51.0	0.002	0.229	0.003
50-59	3.3	4.0	19.0	3.9	4.4	14.6	3.6	4.2	17.5	0.384	0.355	0.173
60-69	3.3	3.5	5.3	3.4	4.0	17.4	3.4	3.8	12.7	0.767	0.25	0.27
70-79	3.3	1.9	-41.5	2.7	4.3	61.6	3.0	3.2	8.4	0.08	0.019	0.63
≥80	3.0	1.9	-38.1	1.8	3.5	94.8	2.4	2.8	17.3	0.522	0.241	0.717
Total	3.6	2.9	-19.5	3.7	4.1	9.2	3.7	3.6	-2.0	0.06	0.288	0.763
Control among treated												
40-49	16.0	3.1	-80.4	11.9	11.7	-2.3	13.6	8.7	-36.0	0.005	0.928	0.056
50-59	9.2	15.5	67.9	8.2	11.8	43.3	8.6	12.8	49.2	0.007	0.012	<0.001
60-69	7.8	11.2	43.9	6.6	9.4	42.8	7.1	10.0	40.8	0.031	0.009	0.001
70-79	7.3	6.5	-11.6	5.1	8.7	72.7	6.1	8.0	30.5	0.683	0.007	0.105
≥80	7.1	11.8	64.7	3.8	9.1	139.0	5.3	9.7	82.6	0.495	0.115	0.157
Total	9.7	10.4	7.1	8.0	10.0	25.0	8.7	10.1	16.3	0.538	0.006	0.021

## Data Availability

The data used to support the findings of this study are available from the corresponding author upon request.
